# 3-(2,3-Dioxoindolin-1-yl)propane­nitrile

**DOI:** 10.1107/S1600536814003985

**Published:** 2014-02-26

**Authors:** Fatima-Zahrae Qachchachi, Youssef Kandri Rodi, El Mokhtar Essassi, Michael Bodensteiner, Lahcen El Ammari

**Affiliations:** a Laboratoire de Chimie Organique Appliquée, Université Sidi Mohamed Ben Abdallah, Faculté des Sciences et Techniques, Route d’Immouzzer, BP 2202 Fès, Morocco; b Laboratoire de Chimie Organique Hétérocyclique, URAC 21, Pôle de Compétences Pharmacochimie, Université Mohammed V-Agdal, Avenue Ibn Battouta, BP 1014, Rabat, Morocco; c X-Ray Structure Analysis Unit, University of Regensburg, D-93053 Regensburg, Germany; d Laboratoire de Chimie du Solide Appliquée, Faculté des Sciences, Université Mohammed V-Agdal, Avenue Ibn Battouta, BP 1014, Rabat, Morocco

## Abstract

The asymmetric unit of the title compound, C_11_H_8_N_2_O_2_, contains two independent mol­ecules (*A* and *B*). Each mol­ecule is build up from fused five- and six-membered rings with the former linked to a cyano­ethyl group. The indoline ring and two carbonyl O atoms of each mol­ecule are nearly coplanar, with the largest deviations from the mean planes being 0.0198 (9) (mol­ecule *A*) and 0.0902 (9) Å (mol­ecule *B*), each by a carbonyl O atom. The fused ring system is nearly perpendicular to the mean plane passing through the cyano­ethyl chains, as indicated by the dihedral angles between them of 69.72 (9) (mol­ecule *A*) and 69.15 (9)° (mol­ecule *B*). In the crystal, mol­ecules are linked by C—H⋯O and π–π [inter­centroid distance between inversion-related indoline (*A*) rings = 3.6804 (7) Å] inter­actions into a double layer that stacks along the *a-*axis direction.

## Related literature   

For biological activity of indoline derivatives, see: Bhrigu *et al.* (2010 ▶); Ramachandran (2011 ▶); Smitha *et al.* (2008 ▶). For similar structures, see: Qachchachi *et al.* (2013 ▶, 2014 ▶).
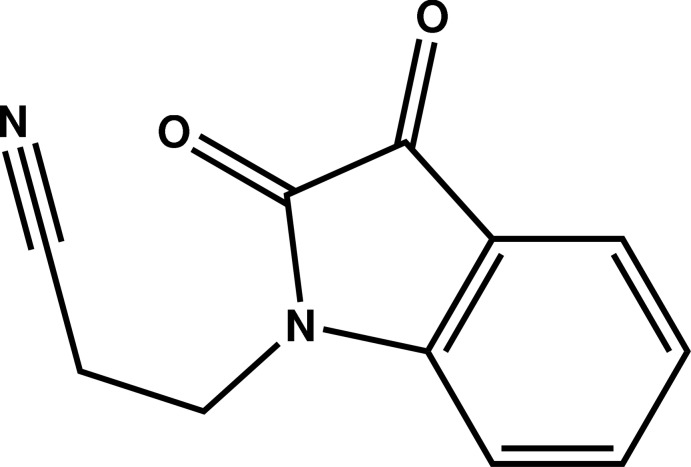


## Experimental   

### 

#### Crystal data  

C_11_H_8_N_2_O_2_
*M* *_r_* = 200.19
Triclinic, 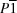

*a* = 7.1967 (2) Å
*b* = 9.9909 (3) Å
*c* = 13.5534 (5) Å
α = 77.508 (3)°
β = 81.551 (3)°
γ = 77.717 (3)°
*V* = 924.44 (5) Å^3^
*Z* = 4
Cu *K*α radiation
μ = 0.84 mm^−1^
*T* = 123 K
0.26 × 0.17 × 0.12 mm

#### Data collection  

Agilent SuperNova (Single source at offset, Atlas) diffractometer
Absorption correction: analytical (Clark & Reid, 1995 ▶) *T*_min_ = 0.827, *T*_max_ = 0.917
5751 measured reflections
3546 independent reflections
3292 reflections with *I* > 2σ(*I*)
*R*_int_ = 0.013

#### Refinement  

*R*[*F* ^2^ > 2σ(*F* ^2^)] = 0.033
*wR*(*F* ^2^) = 0.086
*S* = 1.07
3546 reflections
271 parameters
H-atom parameters constrained
Δρ_max_ = 0.19 e Å^−3^
Δρ_min_ = −0.28 e Å^−3^

### 

Data collection: *CrysAlis PRO* (Agilent, 2013 ▶); cell refinement: *CrysAlis PRO*; data reduction: *CrysAlis PRO*; program(s) used to solve structure: *SHELXS97* (Sheldrick, 2008 ▶); program(s) used to refine structure: *SHELXL97* (Sheldrick, 2008 ▶); molecular graphics: *ORTEP-3 for Windows* (Farrugia, 2012 ▶); software used to prepare material for publication: *WinGX* (Farrugia, 2012 ▶) and *publCIF* (Westrip, 2010 ▶).

## Supplementary Material

Crystal structure: contains datablock(s) I. DOI: 10.1107/S1600536814003985/tk5297sup1.cif

Structure factors: contains datablock(s) I. DOI: 10.1107/S1600536814003985/tk5297Isup2.hkl

Supporting information file. DOI: 10.1107/S1600536814003985/tk5297Isup3.cml

CCDC reference: 988036

Additional supporting information:  crystallographic information; 3D view; checkCIF report

## Figures and Tables

**Table 1:** Hydrogen-bond geometry (Å, °)

*D*—H⋯*A*	*D*—H	H⋯*A*	*D*⋯*A*	*D*—H⋯*A*
C16—H16⋯O1	0.95	2.50	3.2787 (14)	139
C20—H20*B*⋯O1	0.99	2.45	3.4287 (14)	170
C6—H6⋯O4^i^	0.95	2.51	3.1740 (14)	127
C5—H5⋯O3^i^	0.95	2.63	3.5085 (14)	153
C9—H9*B*⋯O1^ii^	0.99	2.49	3.2269 (13)	131

Symmetry codes: (i) 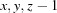; (ii) 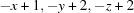.

**Fig. 1.**
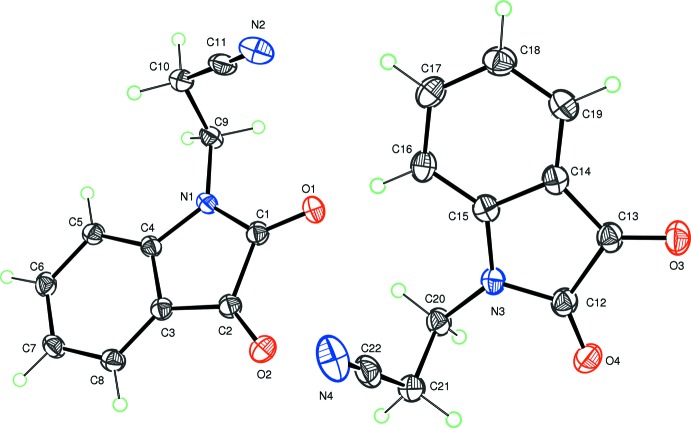
Molecular plot the title compound with the atom-labelling scheme. Displacement ellipsoids are drawn at the 50% probability level.

**Fig. 2.**
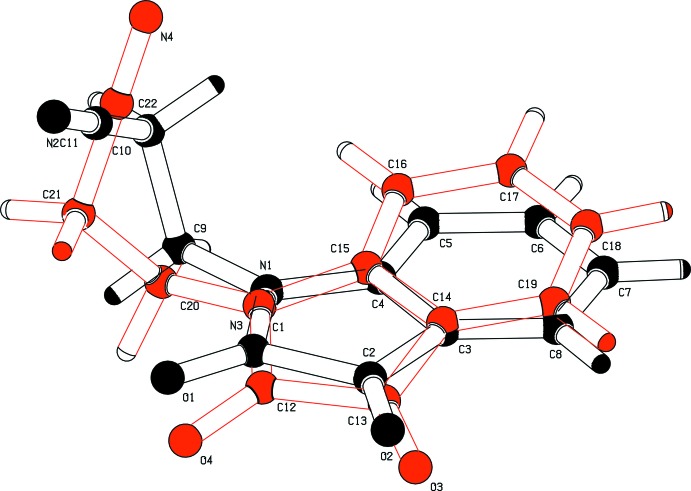
View showing the fitting of the two molecules in the asymmetric unit.

## supplementary crystallographic information

### 2. Structural commentary

Heterocyclic compounds are acquiring more importance in recent years because of their broad pharmacological activities. Isatin and its derivatives are used in organic synthesis and in evaluating new product possessing different biological activities. Isatin derivatives have been reported to show considerable pharmacological actions such as, anti-convulsant, anti-anixity and anti-psychoactives activities (Bhrigu *et al.*, 2010; Ramachandran, 2011; Smitha *et al.*, 2008). As a continuation of our research work devoted to the development of isatin derivatives (Qachchachi *et al.*, 2013; Qachchachi *et al.*, 2014), we report the synthesis of new indoline-2,3-dione derivative by action of alkyl halides to explore other applications.

Each independent molecule of title compound is build up from a fused five- and six-membered rings linked, to a cyano­ethyl chain and to two carbonyl oxygen atoms as shown in Fig. 1. The indoline ring and the two ketonic oxygen atoms are nearly coplanar, with the largest deviation from the mean plane being 0.0198 (9) Å for O1 atom, and 0.0902 (9) Å for the O4 atom, respectively, in the first and second molecule. The fused ring system planes, (N1, C1 to C8) and (N3, C12 to C19), are nearly perpendicular to the mean plane passing through the cyano­ethyl chains (N2, C9–C11 and N4, C20–C22) as indicated by the dihedral angles between them of 69.47 (9) and 69.06 (9)°, respectviely. The two molecules in the asymmetric unit have a similar conformation, except the cyano­ethyl group orientation as shown in Fig. 2.

In the crystal, the molecules are linked by C—H···O hydrogen bonds, Table 1, and π—π inter­actions between the five- and six-membered rings of the N1-containing molecule [inter-centroid distance = 3.6804 (7) Å for symmetry operation: 1-*x*, 1-*y*, 2-*z*] to form double layers that stack along the *a* axis.

### 5. Synthesis and crystallization

To a solution of isatin (0.5 g, 3.4 mmol) dissolved in DMF (30 ml) was added, potassium carbonate (0.61 g, 4.4 mmol), a catalytic qu­antity of tetra-n-butyl­ammonium bromide (0.1 g, 0.4 mmol) and 3-bromo­propane­nitrile (0.3 ml, 3.7 mmol). The mixture was stirred for 48 h; the reaction was monitored by thin layer chromatography. The mixture was filtered and the solvent removed under vacuum. The solid obtained was recrystallized from ethanol to afford the title compound as orange crystals (yield: 52%; M.pt: 383 K).

### 6. Refinement

All H atoms could be located in a difference Fourier map. However, they were placed in calculated positions with C—H = 0.95 Å (aromatic) and C—H = 0.99 Å (methyl­ene), and refined as riding on their parent atoms with *U*_iso_(H) = 1.2 *U*_eq_.

### Crystal data

**Table:**

C_11_H_8_N_2_O_2_	*Z* = 4
*M**_r_* = 200.19	*F*(000) = 416
Triclinic, *P*1	*D*_x_ = 1.438 Mg m^−^^3^
Hall symbol: -P 1	Cu *K*α radiation, λ = 1.54184 Å
*a* = 7.1967 (2) Å	Cell parameters from 4004 reflections
*b* = 9.9909 (3) Å	θ = 4.6–73.3°
*c* = 13.5534 (5) Å	µ = 0.84 mm^−^^1^
α = 77.508 (3)°	*T* = 123 K
β = 81.551 (3)°	Plate, orange
γ = 77.717 (3)°	0.26 × 0.17 × 0.12 mm
*V* = 924.44 (5) Å^3^	

### Data collection

**Table:**

Agilent SuperNova (Single source at offset, Atlas) diffractometer	3546 independent reflections
Radiation source: SuperNova (Cu) X-ray Source	3292 reflections with *I* > 2σ(*I*)
Mirror monochromator	*R*_int_ = 0.013
Detector resolution: 10.3546 pixels mm^-1^	θ_max_ = 73.7°, θ_min_ = 4.6°
ω scans	*h* = −8→8
Absorption correction: analytical (Clark & Reid, 1995)	*k* = −12→9
*T*_min_ = 0.827, *T*_max_ = 0.917	*l* = −16→16
5751 measured reflections	

### Refinement

**Table:**

Refinement on *F*^2^	Primary atom site location: structure-invariant direct methods
Least-squares matrix: full	Secondary atom site location: difference Fourier map
*R*[*F*^2^ > 2σ(*F*^2^)] = 0.033	Hydrogen site location: inferred from neighbouring sites
*wR*(*F*^2^) = 0.086	H-atom parameters constrained
*S* = 1.07	*w* = 1/[σ^2^(*F*_o_^2^) + (0.0448*P*)^2^ + 0.2333*P*] where *P* = (*F*_o_^2^ + 2*F*_c_^2^)/3
3546 reflections	(Δ/σ)_max_ = 0.001
271 parameters	Δρ_max_ = 0.19 e Å^−^^3^
0 restraints	Δρ_min_ = −0.28 e Å^−^^3^

### Special details

**Table:**

Geometry. All e.s.d.'s (except the e.s.d. in the dihedral angle between two l.s. planes) are estimated using the full covariance matrix. The cell e.s.d.'s are taken into account individually in the estimation of e.s.d.'s in distances, angles and torsion angles; correlations between e.s.d.'s in cell parameters are only used when they are defined by crystal symmetry. An approximate (isotropic) treatment of cell e.s.d.'s is used for estimating e.s.d.'s involving l.s. planes.
Refinement. Refinement of *F*^2^ against all reflections. The weighted *R*-factor *wR* and goodness of fit *S* are based on *F*^2^, conventional *R*-factors *R* are based on *F*, with *F* set to zero for negative *F*^2^. The threshold expression of *F*^2^ > 2σ(*F*^2^) is used only for calculating *R*-factors(gt) *etc*. and is not relevant to the choice of reflections for refinement. *R*-factors based on *F*^2^ are statistically about twice as large as those based on *F*, and *R*- factors based on all data will be even larger.

### Fractional atomic coordinates and isotropic or equivalent isotropic displacement parameters (Å^2^)

**Table:**

	*x*	*y*	*z*	*U*_iso_*/*U*_eq_	
C1	0.35182 (15)	0.75842 (11)	1.04434 (8)	0.0189 (2)	
C2	0.27845 (15)	0.61738 (11)	1.07606 (8)	0.0193 (2)	
C3	0.27526 (15)	0.57304 (11)	0.98035 (8)	0.0181 (2)	
C4	0.33583 (14)	0.67561 (11)	0.90158 (8)	0.0171 (2)	
C5	0.34919 (16)	0.66466 (11)	0.80081 (8)	0.0196 (2)	
H5	0.3914	0.7340	0.7475	0.023*	
C6	0.29746 (16)	0.54636 (11)	0.78129 (9)	0.0213 (2)	
H6	0.3044	0.5357	0.7128	0.026*	
C7	0.23621 (16)	0.44382 (11)	0.85865 (9)	0.0218 (2)	
H7	0.2015	0.3652	0.8423	0.026*	
C8	0.22547 (15)	0.45570 (11)	0.95987 (9)	0.0201 (2)	
H8	0.1853	0.3857	1.0133	0.024*	
C9	0.44795 (16)	0.90577 (11)	0.87921 (8)	0.0210 (2)	
H9A	0.5298	0.8784	0.8187	0.025*	
H9B	0.5278	0.9390	0.9189	0.025*	
C10	0.28577 (18)	1.02563 (12)	0.84414 (9)	0.0253 (2)	
H10A	0.3403	1.0976	0.7927	0.030*	
H10B	0.1974	0.9897	0.8113	0.030*	
C11	0.17723 (17)	1.09045 (11)	0.92779 (9)	0.0241 (2)	
C12	0.35805 (16)	0.79086 (12)	1.50595 (9)	0.0231 (2)	
C13	0.30355 (17)	0.93949 (12)	1.53235 (9)	0.0232 (2)	
C14	0.22133 (16)	1.02896 (12)	1.44313 (9)	0.0220 (2)	
C15	0.22224 (15)	0.94421 (12)	1.37283 (8)	0.0204 (2)	
C16	0.15683 (16)	1.00021 (12)	1.27902 (9)	0.0232 (2)	
H16	0.1550	0.9430	1.2317	0.028*	
C17	0.09340 (17)	1.14485 (13)	1.25670 (9)	0.0265 (3)	
H17	0.0477	1.1861	1.1927	0.032*	
C18	0.09507 (18)	1.23038 (13)	1.32516 (10)	0.0285 (3)	
H18	0.0522	1.3283	1.3072	0.034*	
C19	0.15933 (17)	1.17260 (13)	1.41961 (9)	0.0262 (3)	
H19	0.1609	1.2298	1.4670	0.031*	
C20	0.35533 (17)	0.68910 (12)	1.35580 (9)	0.0233 (2)	
H20A	0.4907	0.6454	1.3637	0.028*	
H20B	0.3452	0.7277	1.2827	0.028*	
C21	0.23180 (18)	0.57728 (12)	1.39112 (9)	0.0264 (3)	
H21A	0.2874	0.4972	1.3572	0.032*	
H21B	0.2345	0.5436	1.4653	0.032*	
C22	0.03284 (19)	0.62732 (13)	1.36936 (9)	0.0283 (3)	
N1	0.38031 (13)	0.78390 (9)	0.94098 (7)	0.01830 (19)	
N2	0.09333 (16)	1.14251 (11)	0.99259 (9)	0.0312 (2)	
N3	0.30023 (14)	0.80320 (10)	1.41232 (7)	0.0216 (2)	
N4	−0.12343 (17)	0.66540 (14)	1.35365 (10)	0.0410 (3)	
O1	0.37857 (12)	0.82987 (8)	1.10106 (6)	0.02410 (18)	
O2	0.23689 (12)	0.56414 (9)	1.16318 (6)	0.02605 (19)	
O3	0.33463 (13)	0.96621 (9)	1.61077 (6)	0.0300 (2)	
O4	0.43958 (13)	0.68559 (9)	1.55714 (6)	0.0300 (2)	

### Atomic displacement parameters (Å^2^)

**Table:**

	*U*^11^	*U*^22^	*U*^33^	*U*^12^	*U*^13^	*U*^23^
C1	0.0187 (5)	0.0186 (5)	0.0195 (5)	−0.0013 (4)	−0.0037 (4)	−0.0046 (4)
C2	0.0190 (5)	0.0186 (5)	0.0202 (5)	−0.0023 (4)	−0.0033 (4)	−0.0042 (4)
C3	0.0168 (5)	0.0180 (5)	0.0191 (5)	−0.0017 (4)	−0.0027 (4)	−0.0038 (4)
C4	0.0159 (5)	0.0152 (5)	0.0207 (5)	−0.0010 (4)	−0.0035 (4)	−0.0052 (4)
C5	0.0221 (5)	0.0176 (5)	0.0187 (5)	−0.0024 (4)	−0.0028 (4)	−0.0037 (4)
C6	0.0239 (5)	0.0209 (5)	0.0202 (5)	−0.0003 (4)	−0.0054 (4)	−0.0079 (4)
C7	0.0230 (5)	0.0171 (5)	0.0278 (6)	−0.0029 (4)	−0.0061 (4)	−0.0081 (4)
C8	0.0194 (5)	0.0167 (5)	0.0238 (5)	−0.0030 (4)	−0.0027 (4)	−0.0033 (4)
C9	0.0238 (5)	0.0188 (5)	0.0221 (5)	−0.0083 (4)	0.0004 (4)	−0.0052 (4)
C10	0.0350 (6)	0.0189 (5)	0.0230 (6)	−0.0065 (5)	−0.0061 (5)	−0.0029 (4)
C11	0.0257 (6)	0.0150 (5)	0.0318 (6)	−0.0062 (4)	−0.0057 (5)	−0.0011 (5)
C12	0.0247 (6)	0.0265 (6)	0.0187 (5)	−0.0073 (4)	0.0000 (4)	−0.0044 (4)
C13	0.0240 (6)	0.0278 (6)	0.0195 (5)	−0.0086 (4)	0.0007 (4)	−0.0064 (4)
C14	0.0207 (5)	0.0262 (6)	0.0205 (5)	−0.0072 (4)	0.0005 (4)	−0.0065 (4)
C15	0.0173 (5)	0.0233 (5)	0.0210 (5)	−0.0057 (4)	0.0011 (4)	−0.0053 (4)
C16	0.0199 (5)	0.0300 (6)	0.0204 (5)	−0.0040 (4)	−0.0014 (4)	−0.0072 (4)
C17	0.0213 (6)	0.0315 (6)	0.0242 (6)	−0.0020 (5)	−0.0034 (4)	−0.0024 (5)
C18	0.0253 (6)	0.0238 (6)	0.0347 (7)	−0.0011 (5)	−0.0042 (5)	−0.0049 (5)
C19	0.0247 (6)	0.0264 (6)	0.0293 (6)	−0.0054 (4)	−0.0007 (5)	−0.0103 (5)
C20	0.0259 (6)	0.0241 (6)	0.0208 (5)	−0.0034 (4)	−0.0011 (4)	−0.0083 (4)
C21	0.0359 (7)	0.0226 (6)	0.0217 (6)	−0.0065 (5)	−0.0048 (5)	−0.0042 (4)
C22	0.0360 (7)	0.0307 (6)	0.0232 (6)	−0.0137 (5)	0.0018 (5)	−0.0117 (5)
N1	0.0218 (4)	0.0158 (4)	0.0187 (4)	−0.0050 (3)	−0.0024 (3)	−0.0048 (3)
N2	0.0303 (5)	0.0220 (5)	0.0406 (6)	−0.0057 (4)	0.0009 (5)	−0.0070 (5)
N3	0.0259 (5)	0.0222 (5)	0.0178 (4)	−0.0052 (4)	−0.0020 (4)	−0.0056 (4)
N4	0.0322 (7)	0.0549 (8)	0.0445 (7)	−0.0133 (5)	−0.0001 (5)	−0.0254 (6)
O1	0.0306 (4)	0.0231 (4)	0.0219 (4)	−0.0061 (3)	−0.0050 (3)	−0.0089 (3)
O2	0.0318 (4)	0.0277 (4)	0.0181 (4)	−0.0077 (3)	−0.0017 (3)	−0.0019 (3)
O3	0.0380 (5)	0.0351 (5)	0.0205 (4)	−0.0101 (4)	−0.0033 (4)	−0.0095 (4)
O4	0.0379 (5)	0.0287 (4)	0.0216 (4)	−0.0030 (4)	−0.0056 (4)	−0.0026 (3)

### Geometric parameters (Å, º)

**Table:**

C1—O1	1.2153 (14)	C12—O4	1.2137 (15)
C1—N1	1.3612 (14)	C12—N3	1.3655 (15)
C1—C2	1.5610 (15)	C12—C13	1.5581 (16)
C2—O2	1.2079 (14)	C13—O3	1.2120 (15)
C2—C3	1.4633 (15)	C13—C14	1.4591 (17)
C3—C8	1.3886 (15)	C14—C19	1.3898 (17)
C3—C4	1.4000 (15)	C14—C15	1.4035 (16)
C4—C5	1.3812 (15)	C15—C16	1.3811 (16)
C4—N1	1.4163 (13)	C15—N3	1.4184 (15)
C5—C6	1.3990 (15)	C16—C17	1.3997 (17)
C5—H5	0.9500	C16—H16	0.9500
C6—C7	1.3910 (16)	C17—C18	1.3935 (18)
C6—H6	0.9500	C17—H17	0.9500
C7—C8	1.3919 (16)	C18—C19	1.3880 (18)
C7—H7	0.9500	C18—H18	0.9500
C8—H8	0.9500	C19—H19	0.9500
C9—N1	1.4533 (14)	C20—N3	1.4627 (14)
C9—C10	1.5311 (16)	C20—C21	1.5277 (16)
C9—H9A	0.9900	C20—H20A	0.9900
C9—H9B	0.9900	C20—H20B	0.9900
C10—C11	1.4686 (17)	C21—C22	1.4638 (19)
C10—H10A	0.9900	C21—H21A	0.9900
C10—H10B	0.9900	C21—H21B	0.9900
C11—N2	1.1465 (17)	C22—N4	1.1437 (18)
			
O1—C1—N1	127.39 (10)	O3—C13—C14	131.27 (11)
O1—C1—C2	126.58 (10)	O3—C13—C12	123.77 (11)
N1—C1—C2	106.03 (9)	C14—C13—C12	104.90 (9)
O2—C2—C3	131.42 (10)	C19—C14—C15	121.09 (11)
O2—C2—C1	123.65 (10)	C19—C14—C13	131.24 (11)
C3—C2—C1	104.94 (9)	C15—C14—C13	107.57 (10)
C8—C3—C4	120.97 (10)	C16—C15—C14	121.14 (11)
C8—C3—C2	131.70 (10)	C16—C15—N3	128.32 (10)
C4—C3—C2	107.33 (9)	C14—C15—N3	110.52 (10)
C5—C4—C3	121.79 (10)	C15—C16—C17	117.12 (11)
C5—C4—N1	127.51 (10)	C15—C16—H16	121.4
C3—C4—N1	110.69 (9)	C17—C16—H16	121.4
C4—C5—C6	116.62 (10)	C18—C17—C16	122.25 (11)
C4—C5—H5	121.7	C18—C17—H17	118.9
C6—C5—H5	121.7	C16—C17—H17	118.9
C7—C6—C5	122.30 (10)	C19—C18—C17	120.05 (11)
C7—C6—H6	118.9	C19—C18—H18	120.0
C5—C6—H6	118.9	C17—C18—H18	120.0
C6—C7—C8	120.37 (10)	C18—C19—C14	118.32 (11)
C6—C7—H7	119.8	C18—C19—H19	120.8
C8—C7—H7	119.8	C14—C19—H19	120.8
C3—C8—C7	117.95 (10)	N3—C20—C21	112.93 (9)
C3—C8—H8	121.0	N3—C20—H20A	109.0
C7—C8—H8	121.0	C21—C20—H20A	109.0
N1—C9—C10	113.21 (9)	N3—C20—H20B	109.0
N1—C9—H9A	108.9	C21—C20—H20B	109.0
C10—C9—H9A	108.9	H20A—C20—H20B	107.8
N1—C9—H9B	108.9	C22—C21—C20	113.17 (10)
C10—C9—H9B	108.9	C22—C21—H21A	108.9
H9A—C9—H9B	107.7	C20—C21—H21A	108.9
C11—C10—C9	112.92 (10)	C22—C21—H21B	108.9
C11—C10—H10A	109.0	C20—C21—H21B	108.9
C9—C10—H10A	109.0	H21A—C21—H21B	107.8
C11—C10—H10B	109.0	N4—C22—C21	179.04 (15)
C9—C10—H10B	109.0	C1—N1—C4	111.00 (9)
H10A—C10—H10B	107.8	C1—N1—C9	124.54 (9)
N2—C11—C10	179.16 (13)	C4—N1—C9	124.46 (9)
O4—C12—N3	126.70 (11)	C12—N3—C15	110.66 (9)
O4—C12—C13	127.01 (10)	C12—N3—C20	121.49 (10)
N3—C12—C13	106.28 (10)	C15—N3—C20	126.46 (9)
			
O1—C1—C2—O2	1.10 (18)	C19—C14—C15—N3	177.06 (10)
N1—C1—C2—O2	−179.24 (10)	C13—C14—C15—N3	0.37 (13)
O1—C1—C2—C3	−178.80 (11)	C14—C15—C16—C17	1.18 (16)
N1—C1—C2—C3	0.85 (11)	N3—C15—C16—C17	−177.39 (11)
O2—C2—C3—C8	−0.7 (2)	C15—C16—C17—C18	0.00 (17)
C1—C2—C3—C8	179.16 (11)	C16—C17—C18—C19	−0.66 (19)
O2—C2—C3—C4	179.23 (12)	C17—C18—C19—C14	0.14 (18)
C1—C2—C3—C4	−0.87 (11)	C15—C14—C19—C18	1.04 (18)
C8—C3—C4—C5	−0.18 (16)	C13—C14—C19—C18	176.84 (12)
C2—C3—C4—C5	179.85 (10)	N3—C20—C21—C22	66.72 (13)
C8—C3—C4—N1	−179.42 (9)	O1—C1—N1—C4	179.14 (11)
C2—C3—C4—N1	0.61 (12)	C2—C1—N1—C4	−0.51 (11)
C3—C4—C5—C6	0.55 (16)	O1—C1—N1—C9	−0.68 (18)
N1—C4—C5—C6	179.66 (10)	C2—C1—N1—C9	179.67 (9)
C4—C5—C6—C7	−0.27 (16)	C5—C4—N1—C1	−179.22 (10)
C5—C6—C7—C8	−0.40 (17)	C3—C4—N1—C1	−0.04 (12)
C4—C3—C8—C7	−0.50 (16)	C5—C4—N1—C9	0.60 (17)
C2—C3—C8—C7	179.47 (11)	C3—C4—N1—C9	179.78 (9)
C6—C7—C8—C3	0.77 (16)	C10—C9—N1—C1	−93.32 (12)
N1—C9—C10—C11	69.07 (12)	C10—C9—N1—C4	86.88 (12)
O4—C12—C13—O3	−1.59 (19)	O4—C12—N3—C15	−175.78 (11)
N3—C12—C13—O3	179.97 (11)	C13—C12—N3—C15	2.67 (12)
O4—C12—C13—C14	176.08 (12)	O4—C12—N3—C20	−8.40 (18)
N3—C12—C13—C14	−2.37 (12)	C13—C12—N3—C20	170.05 (9)
O3—C13—C14—C19	2.4 (2)	C16—C15—N3—C12	176.66 (11)
C12—C13—C14—C19	−175.05 (12)	C14—C15—N3—C12	−2.04 (13)
O3—C13—C14—C15	178.60 (12)	C16—C15—N3—C20	10.05 (19)
C12—C13—C14—C15	1.18 (12)	C14—C15—N3—C20	−168.64 (10)
C19—C14—C15—C16	−1.74 (17)	C21—C20—N3—C12	80.57 (13)
C13—C14—C15—C16	−178.43 (10)	C21—C20—N3—C15	−114.16 (12)

### Hydrogen-bond geometry (Å, º)

**Table:**

*D*—H···*A*	*D*—H	H···*A*	*D*···*A*	*D*—H···*A*
C16—H16···O1	0.95	2.50	3.2787 (14)	139
C20—H20*B*···O1	0.99	2.45	3.4287 (14)	170
C6—H6···O4^i^	0.95	2.51	3.1740 (14)	127
C5—H5···O3^i^	0.95	2.63	3.5085 (14)	153
C9—H9*B*···O1^ii^	0.99	2.49	3.2269 (13)	131

Symmetry codes: (i) *x*, *y*, *z*−1; (ii) −*x*+1, −*y*+2, −*z*+2.
